# Comparison of Optical Coherence Tomography Measurement Reproducibility between Children and Adults

**DOI:** 10.1371/journal.pone.0147448

**Published:** 2016-01-25

**Authors:** Ho Kyung Chung, Young Keun Han, Sohee Oh, Seok Hwan Kim

**Affiliations:** 1 Department of Ophthalmology, Seoul National University College of Medicine, Seoul, Korea; 2 Department of Ophthalmology, Seoul National University Boramae Hospital, Seoul, Korea; 3 Department of Biostatistics, Seoul National University Boramae Hospital, Seoul, Korea; Université de Lorraine, FRANCE

## Abstract

**Purpose:**

To compare the reproducibility of SD-OCT (spectral-domain optical coherence tomography) measurements of RNFL (retinal nerve fiber layer) and macular thickness between children and adults.

**Methods:**

Seventy-one eyes of 71 children and 71 eyes of 71 adults were prospectively enrolled. RNFL and macular thicknesses were measured by one operator, with a brief rest between measurements. The two measurements were obtained using the eye tracking and retest function of Spectralis SD-OCT. Reproducibility was evaluated with reference to COVs (coefficients of variation) and ICCs (intraclass correlation coefficients). The ICC values of the RNFL and macular thicknesses were compared, respectively between the two groups, by Fisher’s z-test.

**Results:**

The RNFL and macular thicknesses did not differ between the two groups. The COVs of the RNFL measurements ranged from 0.945 to 4.531% in the children group and from 0.496 to 1.391% in the adults group. In most of the RNFL sectors, the ICCs of the children group (range: 0.731–0.987) were significantly lower than those of the adults group (range: 0.986–0.993). The COVs of the macular measurements ranged from 0.496 to 1.157% in the children group and from 0.275 to 0.656% in the adults group. The ICCs (range: 0.860–0.974) in the children group, significantly lower than for the adults (range: 0.989–0.995), in all of the macular sectors.

**Conclusions:**

The reproducibility of SD-OCT RNFL and macular measurements for children was excellent, albeit statistically lower than that for adults.

## Introduction

Optical coherence tomography (OCT) is a non-invasive cross-sectional imaging technique that allows for high-resolution *in vivo* tissue assessment.[1,2] Since its first introduction in 1991, OCT has been an important macular and retinal nerve fiber layer (RNFL) assessment tool for diagnosis and clinical follow-up of retinal and optic nerve diseases.[3] Time-domain OCT, the first OCT iteration, offers only relatively poor image resolution and requires a long acquisition time, which characteristics limit its utility for pediatrics. The latest version of OCT however, namely spectral-domain OCT (SD-OCT), uses a Fourier-domain interferometric method to provide higher, up to 5 μm resolution.[4] It also enables enhanced image acquisition speeds and eye tracking, advantages which can make it suitable for use with non-compliant pediatric populations.

Recently, SD-OCT has been used in various pediatric retinal and optic nerve applications. Specifically, it can be used to detect subclinical macular abnormalities in retinopathy of prematurity[5] and to investigate anatomical macular and RNFL abnormalities in amblyopic eyes.[6–8] Also, several studies have demonstrated clinical applications of SD-OCT to pediatric ocular diseases including juvenile glaucoma,[9,10] Coats’ disease,[11] and neurofibromatosis.[12]

Before SD-OCT is employed in the clinical pediatric setting, its reproducibility should be validated. A recent study reported good SD-OCT reproducibility for a healthy pediatric population.[13] However, to our knowledge, there has been no direct comparison of SD-OCT reproducibility between children and adults. One SD-OCT modality, Spectralis OCT, has been incorporated into real-time eye-tracking technology as well as software-assisted retest functionality, which applications can reduce noise and enhance reproducibility for poorly compliant pediatric populations. In the present study, we compared children and adult populations in order to evaluate the reproducibility of Spectralis SD-OCT measurements with eye tracking and retest functionality.

## Methods

This prospective observational cross-sectional study was approved by the Institutional Review Board of Boramae Medical Center (16-2013-6). All of the adult participants, along with the parents or guardians of the child subjects, provided written informed consent. The study protocols strictly abided by the guidelines of the Declaration of Helsinki for research involving human participants. All of the children were enrolled through the Myopia Clinic within the Department of Pediatric Ophthalmology of Boramae Medical Center. The adults were enrolled through the Cataract and General Ophthalmology Clinic of the same hospital.

### Subjects

All of the subjects underwent complete ophthalmic examinations, including visual-acuity measurement, intraocular pressure (IOP) measurement by non-contact tonometry, refractive error measurement, anterior segment examination with slit-lamp biomicroscopy, as well as dilated fundus and stereoscopic optic disc examination. The subjects were imaged by fundus photography (TRC-50IX, Topcon, Tokyo, Japan) and SD-OCT (Spectralis; Heidelberg Engineering GmbH). A pupil diameter of at least 4 mm was required for imaging.

The inclusion criteria were as follows: best-corrected visual acuity of 20/30 or better, spherical refraction within ± 6.0 diopters (D), cylinder correction within ± 3.0 D, as well as reliable SD-OCT images (the detailed criteria for SD-OCT reliability are provided below). Subjects were excluded if they had any history of diseases that might cause macular or RNFL damage, diseases that can affect the area where SD-OCT measurements are obtained, amblyopia, IOP greater than 21 mmHg, cup-to-disc ratio greater than 0.6, cupping asymmetry greater than 0.2 or intraocular surgery other than simple cataract extraction. In cases where both eyes were eligible, one eye was chosen randomly for inclusion.

### Spectral-domain optical coherence tomography measurements

All of the SD-OCT images were obtained by a single, well-trained technician using Spectralis SD-OCT (software version 6.0), as described previously.[14] Only good-quality scans with well-focused images, no overt misalignment, no overt decentration of the measurement circle location around the optic disc, a continuous scan pattern without missing or blank areas and a signal strength of better than 20 (40 = maximum) were included in the analyses.

**Fig 1:**
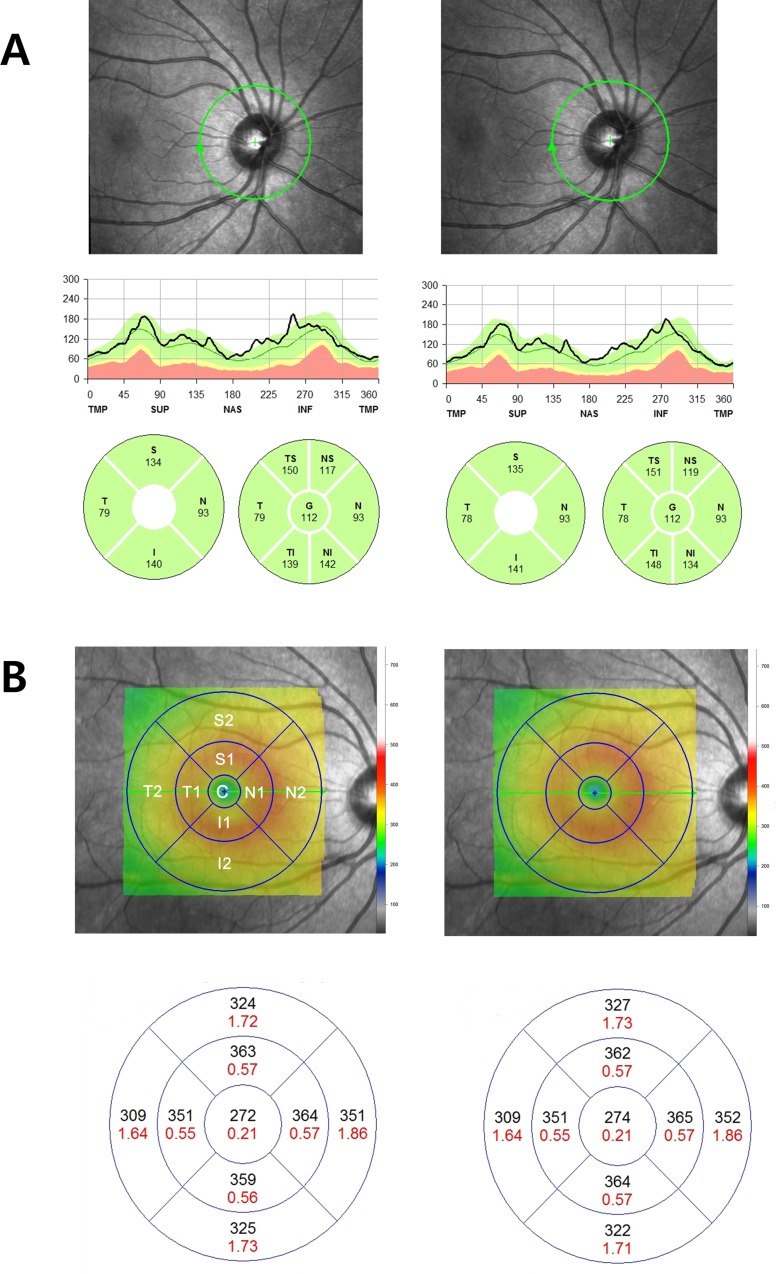
Representative Spectralis SD-OCT scans of (A) retinal nerve fiber layer (RNFL) thickness map and (B) macular thickness map (ETDRS protocol) of normal myopic eye of 9-year-old child. The second scan was obtained with the retest function after a short break. The sectors of RNFL thickness map were designated as a set of S, I, T and N, or a set of TS, NS, TI, NI, T and N. The sectors of the macula thickness map were designated C, S1, S2, T1, T2, N1, N2, I1, and I2.

For RNFL thickness measurements, a scan circle of approximately 3.46 mm diameter was manually positioned at the center of the optic disc while the eye-tracking system was activated. The eye-tracking system incorporates confocal laser scanning ophthalmoscopy enabling capturing of a scanning laser ophthalmoscope fundus image at the same time as the OCT measurement, thereby enabling the system to link every OCT scan to its corresponding position on the SLO (scanning laser ophthalmoscope) fundus image. In the course of RNFL thickness measurement, eye tracking provides for real-time adjustment of the OCT scanner on the simultaneously gathered SLO image, which minimizes motion artifacts, as well as high scanning speed, which reduces the time during which eye movements can occur.[15] For enhanced image quality, Spectralis SD-OCT includes an automatic real-time (ART) function. With ART activated, multiple frames (B-scans) are gathered during the scanning process, and images are averaged for noise reduction.[16,17] In this study, the ART function was set to 100 frames per B-scan for RNFL measurement. The RNFL boundaries underneath the circumpapillary circle were automatically delineated using software algorithms. Then, the RNFL in each image was automatically segmented. In the repeated examination, the retest function, by which the system recognizes the former scanning area on the retina and automatically positions the retest scan at the same location, was activated. Between the two scanning sessions, patients were asked to take a short break and to readjust their head positions. The Spectralis OCT software calculates the average RNFL thickness overall globally (G, 360 degrees), for the four quadrants [superior (S), inferior (I), nasal (N), and temporal (T), each 90 degrees], and then for four additional sectors [i.e., superior-temporal (TS, 45–90 degrees), superior-nasal (NS, 90–135 degrees), inferior-nasal (NI, 225–270 degrees), and inferior-temporal (TI, 270–315 degrees)] (Fig 1A).

For the macular thickness measurements 20 * 20 degree raster scans **(consisting of 25 high-resolution scans)** were performed. The ART function was set to 9 frames per B-scan. An internal fixation light was used to center the scanning area on the fovea while the eye-tracking system was activated. Macular thickness values were calculated for the nine Early Treatment Diabetic Retinopathy Study (ETDRS) areas.[18] An ETDRS plot consists of three concentric rings of 1, 3, and 6 mm diameter. The two outer rings are divided into quadrants by two intersecting lines. Each sector was designated C, S1, S2, T1, T2, I1, I2, N1 and N2, accordingly. (Fig 1B) The ETDRS grid was positioned automatically by the Spectralis OCT software, enabling capturing and extraction of the macular thickness values. No manual adjustments of the grid were performed by the operator. In the repeated examination, the retest function was activated, as explained above with respect to the earlier, RNFL measurement. And, once again, between the two sessions, patients were asked to take a short break and to readjust their head positions.

### Statistical analyses

The baseline characteristics of the study subjects were compared by student t-test for continuous variables and by chi-square test for categorical variables. Coefficients of variation (COVs) and intraclass correlation coefficients (ICCs) were analyzed to determine the reproducibility of the Spectralis OCT measurements. The COV is the standard deviation (SD) divided by the average RNFL thickness of each set, expressed as a percentage. The COVs and ICCs for the RNFL thicknesses were calculated for the global average and for each sector, and those for the macular thicknesses were calculated for each sector. ICCs were obtained while adjusting for spherical equivalent using linear mixed effect model. The ICC values were then compared between the two groups by Fisher’s z-test.[19]

All of the statistical analyses were performed with Statistical Package for the Social Sciences version 21.0 for Windows (SPSS Inc., Chicago, IL, USA) and R version 3.1.2 (http://www.r-project.org, R Foundation for Statistical Computing, Vienna, Austria) with the cocor package. Statistical significance was defined as a *P* value less than 0.05. Bonferroni adjustment was applied for multiple comparisons of the RNFL and macular sector thicknesses.

## Results

On initial assessment, 76 eyes of 76 myopic children and 92 eyes of 92 healthy adults were consecutively enrolled in the present study. Of the 76 children eyes, five (6.58%) were excluded from further analysis due to the unacceptability of their Spectralis SD- OCT scans, as were five (5.43%) of the 92 adult eyes, leaving a sample of 71 eyes of myopic children and 87 eyes of healthy adults. Among those 87 healthy adults, 71 eyes of 71 sex-matched subjects were randomly selected for further analysis.

The baseline characteristics of the study population are summarized in Table 1. No significant RNFL-measurement signal-strength differences were found between the children and adults. The macular-measurement signal strength, meanwhile, was significantly higher in the children group. Also, the spherical equivalent of the children group was significantly more myopic.

**Table 1:** Baseline characteristics of child and adult participants.

	Children	Adults	*P* value
Age (range, yrs)	9.9±1.8 (7–13)	54.8±14.9 (24–80)	<0.001*
Sex	39:32	39:32	1.000^†^
SE (diopters)	-3.82±1.81	-0.52±2.82	<0.001*
Signal Strength			
1^st^ RNFL	27.94±4.47	27.54±3.64	0.551*
2^nd^ RNFL	28.16±4.55	27.66±3.40	0.465*
1^st^ Macula	29.73±4.89	27.76±4.04	0.010*
2^nd^ Macula	29.20±4.12	27.72±4.43	0.041*

SE = spherical equivalent; RNFL = retinal nerve fiber layer. Significant values (*P* <0.05) are in bold.

* Independent t-test

^†^ Chi-square test

The RNFL thicknesses of both groups and their COVs are summarized in Table 2. There were no significant inter-group RNFL thickness differences. The global RNFL thickness had the lowest COV, both in the children group and in the adults group (0.945%, 0.496%, respectively). The highest COV was observed in the nasal sector in both groups (4.531%, 1.391%, respectively). The spherical equivalent-adjusted ICCs of the two groups are provided in Table 3. In all of the RNFL thickness sectors except S and NS, the ICCs of the children group were significantly lower.

**Table 2:** Retinal nerve fiber layer thickness measurements in children and adults.

Parameters	Children			Adults			*P* value*	*P* value^†^
	1^st^ measurement	2^nd^ measurement	COV (%)	1^st^ measurement	2^nd^ measurement	COV (%)		
G	97.41±11.73	96.97±11.25	0.945	97.52±9.83	97.51±9.67	0.496	0.975	0.467
S	119.77±21.45	120.63±22.15	1.632	124.21±15.12	124.32±15.07	0.784	0.215	0.327
T	90.44±20.49	89.16±18.88	1.789	83.65±14.98	83.75±15.05	0.884	0.053	0.082
I	120.18±17.99	119.76±14.86	2.280	120.76±16.03	120.62±16.07	0.720	0.875	0.936
N	59.27±22.61	57.94±19.86	4.531	61.25±14.51	61.04±14.16	1.391	0.566	0.287
TS	142.58±17.62	143.07±17.41	1.697	138.93±17.64	139.28±17.36	0.992	0.172	0.156
TI	144.42±12.34	143.53±12.75	1.757	147.17±17.38	147.28±17.51	0.843	0.479	0.277
NI	98.21±24.08	96.06±21.53	2.332	94.58±19.31	94.15±18.86	0.993	0.191	0.368
NS	97.52±29.35	98.2±30.63	2.499	109.31±16.92	109.3±16.61	1.095	0.006	0.015

COV = coefficient of variation.

*P* value* represents results of t-test between 1^st^ measurement of children group and 1^st^ measurement of adults group. *P*<0.005 (Bonferroni correction for multiple comparisons) was regarded as statistically significant.

*P* value^†^ represents results of t-test between 2^nd^ measurement of children group and 2^nd^ measurement of adults group. *P*<0.005 (Bonferroni correction for multiple comparisons) was regarded as statistically significant.

**Table 3:** Comparison of reproducibility of retinal nerve fiber layer thickness measurements between children and adults.

RNFL	Children		Adults		*P* value*
	ICC	95% CI	ICC	95% CI	
G	0.977	(0.963, 0.985)	0.992	(0.988, 0.995)	**0.001**
S	0.984	(0.975, 0.990)	0.991	(0.985, 0.994)	0.108
T	0.943	(0.910, 0.964)	0.990	(0.984, 0.994)	**<0.001**
I	0.731	(0.601, 0.824)	0.991	(0.986, 0.994)	**<0.001**
N	0.815	(0.718, 0.881)	0.982	(0.972, 0.989)	**<0.001**
TS	0.957	(0.931, 0.973)	0.985	(0.976, 0.991)	**0.002**
TI	0.858	(0.781, 0.909)	0.982	(0.971, 0.989)	**<0.001**
NI	0.946	(0.914, 0.966)	0.992	(0.987, 0.995)	**<0.001**
NS	0.987	(0.979, 0.992)	0.983	(0.972, 0.989)	0.446

ICC = Intraclass correlation coefficient

*Fisher’s z-test. *P*<0.005 (Bonferroni correction for multiple comparisons) was regarded as statistically significant.

The macular thicknesses of both groups and their COVs are listed in Table 4. As was the case for RNFL thickness, there were no significant inter-group macular thickness differences. The T1 thickness had the lowest COV in the children group, and the N1 thickness had the lowest COV in the adults group (0.496%, 0.275%, respectively). The highest COV was observed in C in both groups (1.157%, 0.656%, respectively). The spherical equivalent-adjusted ICCs of the two groups are compared in Table 5. In all of the macular thickness sectors, the ICCs of the children group were significantly lower.

**Table 4:** Macular thickness measurements in children and adults.

Parameters	Children			Adults			*P* value*	*P* value^†^
	1^st^ measurement	2^nd^ measurement	COV (%)	1^st^ measurement	2^nd^ measurement	COV (%)		
C	261.69±20.05	262.41±18.61	1.157	263.80±22.75	263.99±22.85	0.656	0.558	0.653
S1	337.03±17.18	335.94±24.12	0.842	338.77±17.73	338.38±18.09	0.290	0.552	0.497
S2	302.56±18.98	300.94±24.11	1.068	297.80±15.48	298.34±15.77	0.329	0.104	0.447
T1	324.03±15.41	324.03±16.04	0.496	323.92±17.64	323.83±17.78	0.391	0.968	0.945
T2	288.65±19.03	288.72±19.85	0.846	287.75±16.15	287.83±16.48	0.467	0.761	0.772
I1	332.87±15.41	332.14±15.96	0.546	334.92±17.61	335.41±17.34	0.279	0.463	0.245
I2	290.15±20.74	290.39±20.09	0.890	283.06±15.04	282.44±15.04	0.475	0.021	0.009
N1	335.55±16.35	335.32±16.41	0.712	341.76±16.56	342.08±16.68	0.275	0.026	0.016
N2	317.25±19.52	316.10±21.21	0.641	310.97±16.39	310.77±16.22	0.302	0.040	0.095

COV = coefficient of variation.

*P* value* represents results of t-test between 1^st^ measurement of children group and 1^st^ measurement of adults group. *P*<0.005 (Bonferroni correction for multiple comparisons) was regarded as statistically significant.

*P* value^†^ represents results of t-test between 2^nd^ measurement of children group and 2^nd^ measurement of adults group. *P*<0.005 (Bonferroni correction for multiple comparisons) was regarded as statistically significant.

**Table 5:** Comparison of reproducibility of macular thickness measurements between children and adults.

Macula	Children		Adults		*P* value*
	ICC	95% CI	ICC	95% CI	
C	0.952	(0.924, 0.970)	0.989	(0.983, 0.993)	**<0.001**
S1	0.906	(0.853, 0.941)	0.992	(0.987, 0.995)	**<0.001**
S2	0.860	(0.784, 0.910)	0.995	(0.991, 0.997)	**<0.001**
T1	0.970	(0.952, 0.981)	0.989	(0.982, 0.993)	**0.004**
T2	0.973	(0.958, 0.983)	0.991	(0.986, 0.995)	**0.001**
I1	0.971	(0.953, 0.982)	0.989	(0.983, 0.993)	**0.003**
I2	0.974	(0.958, 0.984)	0.995	(0.993, 0.997)	**<0.001**
N1	0.973	(0.958, 0.983)	0.995	(0.991, 0.997)	**<0.001**
N2	0.962	(0.940, 0.976)	0.994	(0.990, 0.996)	**<0.001**

ICC = Intraclass correlation coefficient

^*^Fisher’s z-test. *P*<0.005 (Bonferroni correction for multiple comparisons) was regarded as statistically significant.

## Discussion

Over the last few years, SD-OCT has replaced conventional TD-OCT and become one of the most popular and widely used imaging devices in clinical ophthalmology. SD-OCT has several advantages compared with TD-OCT. Approximately 100-times faster scanning speed, as high as 40,000 A scans/sec, has effectively decreased acquisition time to provide much wider retinal coverage. Moreover, new technology, for example the eye-tracking system, compensates for involuntary eye movements and motion artifacts. By these advances in OCT technology, SD-OCT, significantly, has widened the application for the evaluation of retinal and optic nerve disease to non-compliant young children. However, only a few studies have reported on SD-OCT reproducibility for children. In the present study, we established that the reproducibility of Spectralis SD-OCT RNFL and macular measurements for healthy myopic children was excellent, albeit statistically lower than that in adults, even with eye tracking and the retest function. To our knowledge, this is the first report to directly compare the reproducibility of SD-OCT RNFL and macular measurements between children and adults.

Heidelberg Spectralis OCT software helps to center the optic disc on a frozen fundus image, and uses an integrated eye-tracking system to compensate for eye movement during data acquisition. Additionally, Heidelberg’s noise-reduction technology (the ART function) improves image quality by averaging several consecutive scans and increasing the signal-to-noise ratio[16,17]. Furthermore, the software-assisted retest function, using previous landmarks (re: locations and directions of blood vessels) to recenter the SD-OCT beam on a previously scanned location, facilitates follow-up exams in longitudinal studies. Langenegger et al.[15] showed that the reproducibility of RNFL measurement with Spectralis SD-OCT can be significantly improved by utilizing the eye-tracking system and retest function. In fact, because real-time eye tracking compensates for motion artifacts while the retest function facilitates image acquisition at the same location (which can be especially useful in poorly compliant pediatric populations), Spectralis SD-OCT reproducibility in children might be as good as that in adults. As found in the present study, however, the eye-tracking technology and retest function did not fully compensate for the lower reproducibility resulting from children’s non-compliance, which fact should be considered when interpreting SD-OCT measurements of RNFL and macular thickness for pediatric populations.

Several studies on healthy adults have reported excellent reproducibility of Spectralis OCT RNFL measurement. Wu et al. recorded COVs ranging from 1.45 to 2.59% and ICCs from 0.977 to 0.990.[20] Langenegger et al.’s results, meanwhile, included 1.0–2.5% COVs and 0.97–0.99 ICCs.[15] Serbecic et al. reported intrasessional COVs ranging from 0.545 to 3.97% and intersessional COVs ranging from 0.29 to 1.07% with the retest function.[21] Lange et al. reported 0.63–1.29% COVs and 0.983–0.990 ICCs.[22] Notably, the present study’s adult-eye COVs (0.496–1.391%) and ICCs (0.982–0.992) are comparable to the relevant previous results. In contrast to studies on adult eyes, SD-OCT reproducibility for pediatric populations has only rarely been reported. Altemir et al., having recruited 100 healthy children, recorded COVs of Cirrus OCT RNFL measurement ranging between 2.24 and 5.52%.[13] In the present study, the children-group COVs ranged from 0.945 to 4.531%, and their ICCs, from 0.731 to 0.987, which results are slightly better. However, the reproducibilities of different studies involving different eyes and different study protocols are not directly comparable.

Several studies on healthy adults have reported excellent Spectralis OCT reproducibility of macular thickness measurements by ETDRS area. Bambo et al. reported sectional macular thickness COVs in the 0.61–4.30% range.[23] Pierro et al. recorded Spectralis OCT macular thickness measurement COVs and ICCs ranging from 0.43 to 0.44% and 0.96 to 0.97, respectively,[24] which results are comparable with the present study. Unfortunately, as is the case with RNFL measurement, the macular-measurement reproducibility of SD-OCT has only rarely been reported for children. A recent study found that the COV and ICC of the average macular thickness by Cirrus OCT were 0.97% and 0.942, respectively.[13] In the present study, comparably, we recorded COVs of macular thickness ranging from 0.496 to 1.157% and ICCs from 0.860 to 0.974.

We report herein, for the first time, a lower SD-OCT reproducibility for a pediatric population than for healthy adults, eye tracking and the retest function notwithstanding. Ghasia et al. recently demonstrated a similarity of Spectralis OCT RNFL-measurement reproducibility between children and healthy adults.[25] However, they evaluated only 12 normal adults and 13 normal children, and did not perform any segmental analysis of the RNFL and macula. Our study, by contrast, recruited substantial pediatric and adult subject populations and performed segmental analysis.

Among our results, the macular-measurement signal strength was significantly higher for children than for adults. The several adult participants with mild cataract could have influenced this result; nonetheless, the RNFL-measurement signal strength did not differ between the two groups. Unlike the case with macular measurement, when RNFL thickness was measured, the fixation target moved nasally. Therefore, the signal strength of the RNFL measurements might have been affected by the relatively poor cooperation of the child participants in fixating the nasally displaced target.

Our study has several limitations. First, we recruited the pediatric population from a myopia clinic within a pediatric ophthalmology department. Consequently, the two groups’ spherical equivalents were significantly different. Myopia can affect RNFL and macular thickness measurements, especially in high myopia. In our study, however, the average myopia of the children was -3.8 diopters, which represents only mild-to-moderate myopia, and we excluded patients with high myopia (< -6 Diopter) from the enrollment. Additionally, and notwithstanding the refractive difference between the children and adult groups, there was no inter-group RNFL or macular thickness difference (Tables 2 & 4). Supporting our contention is a recent study by Youm et al.[26], which showed that myopia did not affect the reproducibility of RNFL or macular thickness measurement. The aim of our study, in fact, was to evaluate and compare OCT-measurement reproducibility between children and adults, not thickness measurement *per se*. Also, to minimize the possible effect of myopia-related bias (if any) on OCT-measurement reproducibility, we adjusted for any myopia effect by linear mixed effect modeling and compared the obtained ICCs. On this basis, we are confident that childhood myopia did not affect the children/adult OCT-reproducibility comparison in our study. Second, this study included only healthy eyes except mild to moderate myopia. Among eyes with macular or optic nerve disease, reproducibility findings might differ. Third, we evaluated only intra-visit reproducibility; we did not consider inter-visit reproducibility. Further study with an adjusted design and a larger subject population is warranted.

In conclusion, we showed that Spectralis OCT, utilizing eye-tracking and the retest function, can measure RNFL and macular thickness in a child population with excellent reproducibility. However, the reproducibility for the adult population was higher still. As SD-OCT has widened the application for the diagnosis and monitoring of pediatric ocular disease, it should be considered when interpreting SD-OCT measurements for pediatric populations.
